# Tensor Network State Algorithms on AI Accelerators

**DOI:** 10.1021/acs.jctc.4c00800

**Published:** 2024-10-14

**Authors:** Andor Menczer, Örs Legeza

**Affiliations:** † Strongly Correlated Systems “Lendület” Research Group, Wigner Research Centre for Physics, H-1525 Budapest, Hungary; ‡ Eötvös Loránd University, Pázmány Péter Sétány 1/C, 1117 Budapest, Hungary; § Institute for Advanced Study, Technical University of Munich, Lichtenbergstrasse 2a, 85748 Garching, Germany

## Abstract

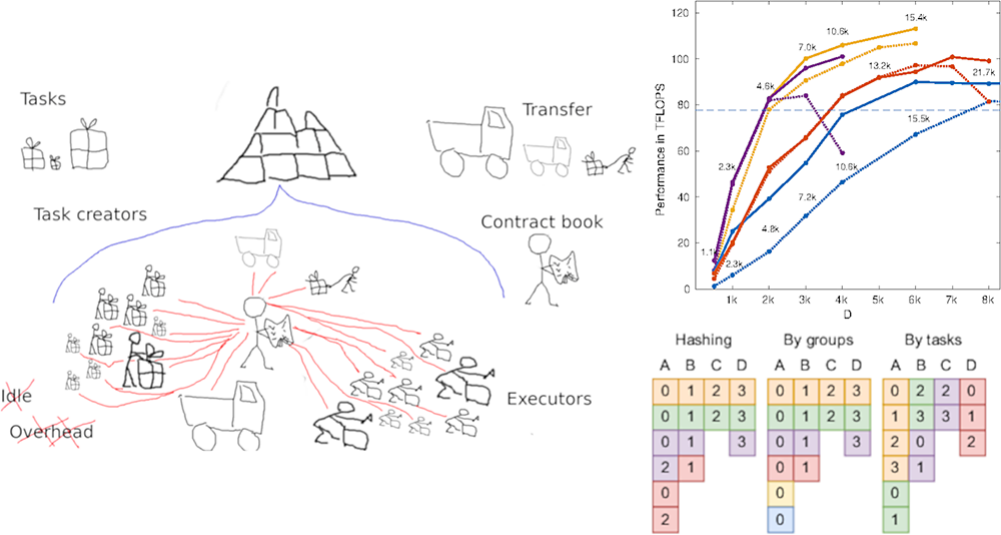

We introduce novel algorithmic solutions for hybrid CPU-multiGPU tensor network state algorithms utilizing non-Abelian symmetries building on AI-motivated state-of-the-art hardware and software technologies. The presented numerical simulations on the FeMo cofactor, which plays a crucial role in converting atmospheric nitrogen to ammonia, are far beyond the scope of traditional approaches. Our large-scale *SU*(2) spin adapted density matrix renormalization group calculations up to bond dimension *D* = 2^16^ on complete active space (CAS) size of 18 electrons in 18 orbitals [CAS(18, 18)] demonstrate that the current limit of exact solution, i.e. full-CI limit, can be achieved in fraction of time. Furthermore, benchmarks up to CAS(113, 76) demonstrate the utilization of NVIDIA’s highly specialized AI accelerators via NVIDIA Tensor Cores, leading to performance around 115 TFLOPS on a single node supplied with eight NVIDIA A100 devices. As a consequence of reaching 71% of the full capacity of the hardware, the cubic scaling of computational time with bond dimension can be reduced to a linear form for a broad range of *D* values; thus, breaking the current computational limits of small CAS spaces in ab initio quantum chemistry and material science is becoming a reality. In comparison to strict *U*(1) implementations with matching accuracy, our solution has an estimated effective performance of 300–500 TFLOPS, which emphasizes the mutual need for both algorithmic and technological developments to push current frontiers on classical computation.

## 1 Introduction

There is an ever growing demand for efficient simulation of quantum systems via classical computation.^1^ Despite the industry’s best effort to create increasingly more powerful hardware,^2−8^ a fundamental limitation is known for a long time: the so-called curse of dimensionality, that is, the computational effort and the dimension of the Hilbert space for systems described by multiparticle Schrödinger equation scale exponentially with the number of constituents.^9^ Therefore, searching for algorithmic solutions with the potential to reduce the exponential scaling by controlled approximations and doing so in a way that modern High-Performance Computing (HPC)^10^ infrastructures are fully taken advantage of, is in the focus of modern quantum physics and chemistry.^11−21^

The density matrix renormalization group (DMRG) method,^22^ a subclass of tensor network state (TNS) methods, has the potential to fulfill both criteria as the number of required arithmetic calculations can be reduced by multiple magnitudes. In consequence, the exponential running time collapses into polynomial complexity.,^23−32^ In addition, large-scale tensor operations can be substituted with multimillion vector and matrix operations, leading to layers of abstractions ranging from low level SIMD^33^ instructions to high level HPC scheduling.

Quite recently, an efficient hybrid CPU-multiGPU implementation of the DMRG method for complex chemical systems has been introduced^21^ demonstrating a linear scaling in performance with the number of GPU devices on a single node, achieving a dramatic decrease in computational time. Although, the theoretical FP64^34^ performance ceiling without tensor cores has been reached on a single node supplied with eight NVIDIA A100 devices, even further computational power can be utilized on such state-of-the-art hardware due to AI-motivated technology. In fact, the tremendous developments in IT technology, like NVIDIA’s latest Grace Hopper and Blackwell hardware platforms (not utilized in the current work), provides such an increase in computational power that is hard to even imagine.^35^ Therefore, it is the main aim of our current work to introduce novel algorithmic solutions that are fully scalable on HPCs and can exploit the benefits of AI accelerators together with non-Abelian symmetries to boost performance tremendously. Consequently, the cubic scaling of computational time with the so-called bond dimension, the main algorithmic parameter determining numerical accuracy, can be reduced to a linear form for a broad range of values.

Our in-house developed hybrid CPU-multiGPU solution relies on the new and improved main pillars:

1. A formulation of the mathematical framework where the non-Abelian symmetry related tensor algebra^36−50^ based on Wigner-Eckhart theorem^51^ is fully detached from the conventional tensor network layer, so that massively parallel matrix and tensor operations can be performed without additional overheads. For further details we refer to Sections 2.2 and 2.3.
2. Custom tailored virtual memory management which ensures data is produced with high spatial locality, which together with the use of specific sequences of strided batched matrix operations translates to significantly higher overall throughput. For further details we refer to Section 3.1.
3. A new model for host side MIMD^52^ parallelization, where scheduling is decentralized, threads are autonomous and interthread communications are solely limited to interactions with globally visible lock-free constructs. Such a design promises similar thread behavior to professional human players in team sports, that is, each person makes decisions based solely on one’s own perceived environment, without feeling a necessity to verbally discuss the next course of action. Despite the team’s lack of explicit communication and reliance on external decision making, such a group of individuals can play the game with perfect team synergy. For further details we refer to Sections 3.2, 3.3, and 3.4.
4. An adaptive buffering technique that dynamically matches the base unit of cache repositories to temporally available system resources. By doing so we maximize overall performance by minimizing locally occurring IO overheads. For further details we refer to Sections 3.5 and 3.6.

All of these are necessary for not only reaching, but breaking the theoretical upper limit of conventional GPU accelerated computing. Only when also accounting for the AI specific performance boosting circuitry called NVIDIA tensor core units could we fit our measured performance within theoretical boundaries. The resulting *SU*(2) spin adapted CPU-multiGPU version of the DMRG can be applied directly to many problems in quantum chemistry, condensed matter physics and nuclear shell models.^29,41,43,53^

The paper is organized as follows. In Section 2 we present a brief overview of non-Abelian symmetry in the context of tensor network state methods highlighting those technical aspects which are relevant for efficient parallelization. In Section 3 we introduce methods developed according to various parallelization strategies and novel algorithmic solutions to achieve an efficient hybrid CPU-multiGPU kernel for simulations via non-Abelian symmetries. In Section 4 we present numerical benchmark results and scaling analysis for selected chemical systems together with discussions on future possibilities. Point-by-point conclusions, Section 5, close our presentation. Technical details of the derivation of the Wigner-9j function is collected in the Supporting Information (SI).

## 2 Theory

In this section we discuss non-Abelian symmetry in the context of tensor network state methods and focus only on those aspects that are relevant for efficient implications on HPC infrastructures. Our algorithmic developments is presented for the density matrix renormalization group (DMRG) method^22^ that is a special variant of tensor network state (TNS) algorithms.^24,27−30,32^ We focus on a very general form of the Hamiltonian operator, implemented in our code,^54^ that can treat any form of nonlocal interactions related to two-particle scattering processes. The corresponding Hamiltonian can be written in the form
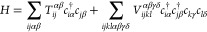
1where the indices α, β, γ, δ label internal degrees of freedom, like spin or isospin. The operators *c*_*i*α_^†^ or *c*_*i*α_ usually denote spin ladder or Fermion creation and annihilation operators. Indices *i*, *j*, *k*, *l* label, in general, arbitrary modes which allows us to simulate strongly correlated quantum many body problems in various fields of disciplines, like condensed matter physics, nuclear structure theory or quantum chemistry even in the relativistic domain.^22,53,55−61^ In quantum chemistry applications, modes are orbitals, but even there is a large freedom finding the optimal representation, for example, via Fermionic mode optimization.^62−64^

The related eigenvalue problem is obtained by the iterative diagonalization of the so-called effective quantum many body Hamiltonian^28^ via the Lánczos or Davidson algorithms corresponding usually to 85–90% of the total execution time. In the TNS/DMRG methods, renormalized block operators are formed via the course of the network contraction procedure which is responsible for another 5–10% of the total execution time. The underlying TNS/DMRG tensor and matrix algebra can be organized into several million of independent operations (tasks) and performed in parallel according to the so-called quantum number decomposed representations. The size of the full matrices, denoted as DMRG bond dimension, *D*, determines the accuracy of the calculations and at the same time the required computational complexity. The overall scaling of the DMRG for models described by eq 1 is ) where *N* stands for the number of modes, i.e., for the system size. The memory requirement is proportional to *D*^2^*N*^2^. We guide the readers to ref (65). for a recent numerical scaling analysis, while further details of the algorithm can be found in various review articles.^23,24,27−30,32^

In our implementation, the non-Abelian symmetry related complex mathematical models based on precalculated data structures, that are computationally lightweight as no actual data transformation occurs, are handled via MATLAB^66^ In addition, since GPU devices are handled at a lower abstraction level, all mathematical aspects of the DMRG algorithm is considered on the host (CPU) side. In contrast to this, the entire number crunching computational part is implemented in native C++, and GPU devices are used only for executing basic algebraic operations in large batches. In the following, we discuss only those algorithmic developments which are relevant for boosting the performance via non-Abelian symmetry and exploiting the computational power of the underlying AI accelerators, while for further details on our hybrid CPU-multiGPU kernel we guide the readers to ref (21).

### 2.1 Hierarchy of Tasks

In DMRG the modes of a network are partitioned into subsystems (blocks) and for efficient treatment of long-range interactions, precontracted operators are formed to reduce the computational complexity from *N*^4^ to *N*^2^.^55,56^ The Hamiltonian in eq 1 is constructed from these operators using the matrix product operator (MPO) representation.^23,24,28,29,67,68^ In the two-site DMRG topology the related matrix vector multiplications in the iterative diagonalization via the Davidson or Lánczos algorithm is obtained from an accumulated sum of four matrix multiplications along four distinct dimensions of a four-dimensional tensor, i.e.,
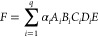
2where *q* stands for the number of independent operator combinations in the MPO, α_*i*_ is a precalculated constant, *A*_*i*_, *B*_*i*_, *C*_*i*_ and *D*_*i*_ label operators acting on the four subsystems, and *E* and *F* are the 4-index tensor representations of the quantum many body wave function. In our implementation, each of such operator combination is stored in a table (operator-table). As an example, SI presents a brief overview of the structure of the operator-table for the kinetic term in eq 1.

In the TNS/DMRG algorithm, the matrices and tensors are decomposed into smaller components (sectors) based on quantum numbers, i.e., a full matrix is stored according to row-column quantum number sector pairs (sector-table) and the corresponding dense matrix.^13,15,21,29^ All operations of the underlying DMRG linear algebra is developed via such sector representation by storing sector combinations in lookup tables (sector-task-tables). For example, for a given *i* in eq 2 the algebra is decomposed into a series of elementary tensor operations, where sector components of the matrices and tensors are addressed by six integer pointers, *p*_*n*_^K^ with *K* ∈{*A*, *B*, *C*.*D*, *E*, *F*}, stored in rows of the sector-task-table having as many rows, χ_*i*_, as the given operation would decompose into, i.e.,
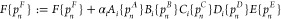
3with *n* = 1, ···, χ_*i*_.

For long-range interactions, the contracted block operators are position dependent, thus in our tensor library,^69^ the sector dependent dense matrices of the same operator type have a third index as well, labeling their positions in the given subsystem. These form contiguous segments in memory, while operations often access only subsets of them which requires clever on-the-fly regrouping in order to achieve an efficient and highly parallel execution of the given algebra also being capable of utilizing AI accelerators. Therefore, a key point of our solution is that we aim to avoid moving data additionally on the GPUs by filling the GPU global memory in such a way that it already conforms to all criteria of strided batched matrix operations. This will be discussed in detail in Section 3.

Therefore, in our implementation, each operator combination of eq 2 is stored in an operator-table and each operator combination is supplied with the corresponding sector-task-table. As a result, there are three main loops that must be executed: loop over the rows of the operator-table, for each row a loop over the rows of the corresponding sector-task-table and finally a loop over the position index within the subsystem blocks. The number of all elements resulting from the three nested loops provides the number of independent operations (tasks) that must altogether be executed to perform one Lánczos/Davidson iteration step.^13^ Since the sector sizes and position ranges are available a prior execution, the computational complexity can be precalculated and various dynamic scheduling algorithms can be developed optimizing overheads. As discussed in ref (21), there are multiple methods for organizing the underlying DMRG algebra into independent tasks, with each varying in asymptotic space, IO time and compute time complexities. In Section 3 we present the importance of task hierarchy, i.e., how to group subsets of tasks together at different abstraction levels in order to maximize performance and minimize IO overhead. This is an important step for GPU accelerated DMRG calculations on model Hamiltonians with long-range interactions as they require large amount of stored data that far exceeds current GPU memory sizes.^13,21^^70^ As a result, a significant portion of data is not locally available for all devices at all times, thus an efficient algorithm for handling the transfer of the required slices of data from a suitable source to a suitable destination at a suitable time becomes mandatory.

### 2.2 Utilization of Non-Abelian Symmetry

In case of *SU*(2) symmetry the matrix and tensor algebra in TNS/DMRG can be performed just like for *U*(1) symmetry except that the Hamiltonian is reformulated in terms of so-called reduced operators.^36−38^ These operators have smaller dimensions compared to the original operators defined for *U*(1) symmetry. For example, for a spin-1/2 Fermion model given by eq 1 in case of *SU*(2) spin symmetry out of the four local basis expressed in occupation numbers together with spin degrees of freedom, |0⟩, |↓⟩, |↑⟩|↑↓⟩ only three remain as the |↓⟩ and |↑⟩ are connected via the *SU*(2) spin symmetry. In certain special circumstances, when *SU*(2) charge symmetry is also applied, the dimension of the local Hilbert space shrinks to two as bases |0⟩ and |↑↓⟩ also transform to each other.^38,39^ Accordingly, reduced operators with dimension three or two are formed via the Wigner-Eckhart theorem^51,71^ and after the related rescaling of the couplings constants in eq 1 the non-Abelian version of the Hamiltonian is achieved.^36,38,41,44,45^ The tensor and matrix algebra, however, requires further corrections as reduced operators also possess quantum numbers, that are invariant under transformation of the given non-Abelian symmetry.^51,71^ The appearing scalar multiplication factors are determined by the spin of the bra and ket states, by the spin of reduced operators appearing in the given operation and by the corresponding Clebsch-Gordan coefficients.^71^ Here we summarize only those formulas that are used in the context of the current work using a Matlab implementation of the so-called Wigner-9j and Wigner-6j formalism^72−74^ while further details can be found in the original publications.^36,38−50^

More precisely, when matrix elements are calculated for a tensor product operation between bra and ket states their total spin *j* and the internal degrees of freedom, – *j* ≤ *m* ≤ *j*, must also be taken into account. In addition, tensor product of basis states with *j*_1_ and *j*_2_ quantum numbers can generate matrix elements with quantum number in the range of |*j*_1_ – *j*_2_|≤ *j* ≤ *j*_1_ + *j*_2_. Similar arguments hold for operators with spin *k*_1_ and *k*_2_, i.e., the resulting operator can have spin |*k*_1_ – *k*_2_|≤ *k* ≤ *k*_1_ + *k*_2_. To fix notations, we consider a tensor product operation where operators have spin *k*_*i*_ and states are labeled as |*j*_*i*_, *m*_*i*_⟩ with *i* ∈{1, 2}, i.e.
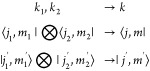
4The corresponding scalar factor that modifies the conventional tensor product operation developed for *U*(1) symmetry, *C̃*, can be determined for non-Abelian *SU*(2) symmetry as
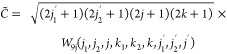
5where the detailed derivation of *W*_9j_ is collected in the SI (see eqs S2–S8). As a result of the Wigner-Eckhart theorem, the underlying TNS/DMRG matrix and tensor algebra can be reformulated according to the non-Abelian quantum numbers. Therefore, instead of keeping renormalized states corresponding to *U*(1) symmetries, they are selected according to quantum numbers of multiplets.^37,38^ Usually a factor of 2 to four reduction in bond dimension can be achieved for the same accuracy which can lead easily to an order of magnitude speedup as will also be demonstrated in Section 4.

### 2.3 Required Modification for Non-Abelian Symmetry and Efficient Implementation on HPC Infrastructure

For models in which the local Hilbert spaces of the two-intermediate modes are decomposed into one-dimensional sectors based on quantum numbers, like in the model systems studied in this paper, the complexity of eq 2 can be reduced to a series of matrix multiplications by preprocessing the corresponding sector-task-table based on eq 3, and storing the product of the sector contributions of *B*_*i*_{*p*_*n*_^B^}, *C*_*i*_{*p*_*n*_^C^} and α_*i*_ in an additional table (sector-value-table).^21^ This is advantageous when operations given by eq 2 are executed several times, for example, in case of the diagonalization procedure. Using the formalism outlined by eqs S2–S8 the Clebsch-Gordan layer can be separated from the MPS layer (see also refs (47 and 49)) leading to an additional scalar multiplication only for both the matrix and tensor product operations. Preprocessing a given sector-task-table not only provides pointer structures addressing proper sector components of the operators *A*_*i*_, *B*_*i*_, *C*_*i*_, *D*_*i*_, *E* and *F* in eq 2, but also gives the corresponding quantum numbers. These can be used immediately to precalculate the corresponding scalar factors derived in Section 2.2 to include contributions of the Clebsch-Gordan layer and modify the related entries in the sector-value-table.

For example, the tensor product operation, *A* ⊗ *B*, in case of *SU*(2) spin symmetry is performed like in case of *U*(1) symmetry, i.e., tensor products from sector components of *A* and *B* are formed by processing the corresponding sector-task-table, but each result is multiplied with an additional scalar given by eq 5. The scalar corrections, *C̃*, for tensor and matrix operations in eq 3 can be determined similarly. However, the *W*_9*j*_ in eq 5 is a complicated function and a computationally demanding object, thus for an efficient implementation it is mandatory to precalculate and store its matrix elements. Since the size of such an object can be very large, even exceeding the available RAM size for a given problem, it is split up into three smaller components, that we label as *W*_9*j*_^(1)^, *W*_9*j*_^(r)^ and *W*_9*j*_^(h)^. Assuming blocks with highest spin value *S*, the intermediate site with *s*, and operators with spin *S*_O_ all components of the *W*_9*j*_^(1)^ tensor are precalculated for index ranges , , , , , , *j* ∈ {|*j*_1_ – *j*_2_|, ···, *j*_1_ + *j*_2_}, *j′*∈ {|*j*_1_*′*– *j*_2_*′*|, ···, *j*_1_*′* + *j*_2_*′*}, and *k* ∈ {|*k*_1_ – *k*_2_|, ···, *k*_1_ + *k*_2_} and stored. Practically, for each *j*_1_, *j*_2_, *k*_1_, *k*_2_, *j*_1_*′*, *j*_2_*′* index-tuple a 3-dimensional matrix is stored, i.e. it has the size of
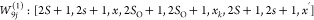
6where *x*, *x*_*k*_, *x′* is determined by the other six indices. The *W*_9*j*_^(1)^ tensor can be used as a lookup table for the renormalization step when tensor product of the block and the site is formed. Similarly, when the tensor product of the site and block is formed the non-Abelian symmetry related scalar factors are taken from *W*_9*j*_^(*r*)^ that is precalculated and stored in the form:
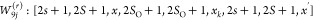
7The scalar factors for each operation in eq 3 is modified with corresponding components of *W*_9*j*_^(1)^, *W*_9*j*_^(r)^, and *W*_9*j*_^(h)^. Here, *W*_9*j*_^(h)^ is an object with size
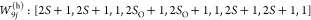
8where *j* = *j′* = *S*_TG_ as the spin of the target state is fixed, and *k* = 0 as the Hamilton is a spin zero object. The precalculation of *W*_9*j*_^(1)^ and *W*_9*j*_^(r)^ is computationally demanding while *W*_9*j*_^(h)^ is a small object and calculated for a given target state at the beginning of the DMRG calculation. If data set stored in *W*_9*j*_^(1)^ or *W*_9*j*_^(r)^ is overindexed via the course of the calculations the required tensor element is calculated and the W tensor is updated. Choosing, for example, *S* = 19/2, *s* = 2, *S*_O_ = 2 we have not experienced such scenario for problems studied in this work and calculating *W*_9*j*_^(1)^ and *W*_9*j*_^(r)^ takes about three minutes while storing requires only 2 × 2.1 MB disk space in a compressed file format.

Having *W*_9*j*_^(1)^, *W*_9*j*_^(r)^ and *W*_9*j*_^(h)^ in hand, in a given DMRG iteration step for each entry of the operator-table the supplied sector-task-table is preprocessed and sector and operator dependent non-Abelian scalar correction factors are determined and stored in the sector-value-table. This means that actual execution of the independent tasks given by the tables follows only after such initialization procedure is completed, which makes our implementation ideal for HPC infrastructures via dynamic scheduling protocols even in case of non-Abelian symmetries. Although, in our discussion we have restricted ourselves to a single non-Abelian symmetry and only for the *SU*(2) spin symmetry, the presented algorithmic solution works in general^49,50^ once the proper mathematical treatment of the given symmetry is implemented. In the current work, we use *U*(1) charge symmetry and *SU*(2) spin symmetry.^38^

## 3 Algorithmic Solutions

In this section, we present details of the various algorithmic solutions of our new model of parallelization that are key to boost the performance for non-Abelian symmetries and utilizing AI accelerators.

### 3.1 Partial Strided Batched Matrix Multiplication for Summation

**Figure 1:**
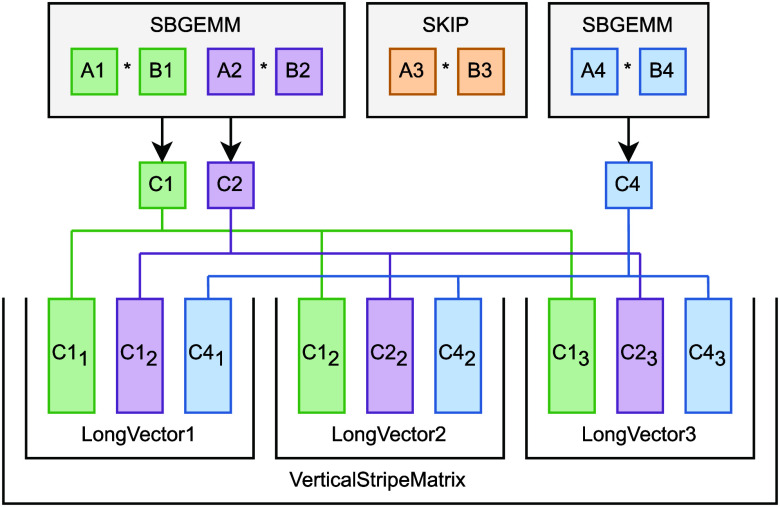
Example for Partial SBMM4S. Input matrices are stored consecutively in memory, however for algorithmic reasons some multiplications can be skipped. Because of this, the multiplication has to be broken up into multiple SBGEMM operations. The leading dimensions and stride values are set in a way that the vectors of the result matrices became interleaved. Since the vectors from different result matrices are next to each other in memory, each vector group can be reinterpreted as a single long vector. Similarly, the sequence of such vectors can be viewed as an either horizontally or vertically very long stripe-like matrix. Using such a matrix as the left or right operand for an ordinary matrix multiplication will work the same way as it does for regular SBMM4S: the entire operation takes only a single kernel call with no additional reduction step needed. Please note that the construction of long vectors and the stripe matrix are handled by the SBGEMM operations, thus they pose no additional overhead.

Unlike prior designs, we no longer treat the multiplications of matrix arrays as a sequence of GEneric Matrix Multiplications (GEMMs) or as a singular Strided Batched GEMM (SBGEMM) operation, but rather as a sequence of SBGEMMs. This enables us to exert more finesse over the grouping of same-sized matrices. In particular, when a matrix array is deemed unfit for concurrent multiplication, it might still be possible to create smaller, SBGEMM compatible batches within the array (Figure 1).

By decomposing the matrix arrays into such subgroups and dealing with each of these with a single SBGEMM, the overall multiplication of the entire matrix array will yield significantly higher parallelization compared to sequential GEMMs as long as the matrices residing in these subgroups can be numerous. In other words, instead of interpreting matrix arrays as either Single-Instruction Multiple-Data (SIMD) batches that are compatible with single SBGEMM operations or Multiple-Instruction Multiple-Data (MIMD) batches that are compatible with sequences of GEMMs, we interpret all matrix arrays as MIMD batches of SIMD sub-batches compatible with sequences of SBGEMMs.

This is not an alternative solution, but a more general approach, because in special cases—by restricting the size of the MIMD batch or all SIMD sub-batches to a single element—we end up with either a single SBGEMM operation or a sequence of one matrix long SBGEMM operations, which are effectively just ordinary GEMMs.

Based on whether matrices are stored in column-major or row-major format, consecutively stored matrices can be reinterpreted as a single matrix with a magnitude higher column or row count. Such a matrix will take shape as either a horizontally or vertically very long stripe-like structure. In order to make standard matrix multiplication possible, a horizontally stacked stripe needs to be paired with a vertically stacked one made from the same number of matrices. In both column-major and row-major formats, one of these two operands are naturally available as long as the building blocks (the matrices) are stored consecutively in memory. The other operand can be created through proper usage of leading dimensions and stride value of SBGEMM operations determining memory offsets as explained in detail in ref (21) including pseudocodes as well.

During matrix multiplication, the dimensions of the resulting matrix is defined by the rows of the left operand and columns of the right, and as such, the result from the multiplication of two compatible stripe matrices have the same dimensions as the multiplicative result of its building blocks. Multiplying *m* by *k* sized matrices with *k* by *n* sized ones a total of *c* times is equivalent in terms of computational complexity to multiplying a single *m* by *k* ∗ *c* horizontal stripe matrix with a *k* ∗ *c* by *n* vertical stripe matrix. However, in the latter case the results from elementary multiplications are added to the same output matrix, thus no additional reduction step is necessary.

Please note that while sequentially executed GEMM operations can be directed into the same output matrix, making the output a sum of all prior multiplications, batched GEMM operations require the result matrices to not overlap. In the end, with traditional GEMMs or SBGEMM we either give up parallelism or create additional overhead in the form of having to reduce multiple results into one as a last step. By utilizing stripe matrices multiple matrix multiplications can be merged into a single kernel without creating an unnecessary reduction step.

Stripe matrices can be created independently. Furthermore, as the batch of batched matrix operations create the same stripe matrix, regardless of how the matrices are organized into SBGEMM groups, different batches corresponding to different stripe matrices can be grouped independently. This level of freedom holds true for the final GEMM operations as well. In other words, merging two stripe matrices through multiplication can be done via a single GEMM operation or can be broken up into multiple GEMM operations in case the naturally occurring stripe matrix is segmented (not contiguous in memory).

### 3.2 Contractor Threads

In theory, perfect software scalability is attainable via static scheduling if the executable tasks are independent, same-sized and the program can produce an arbitrary number of these tasks. With such constraints, the real world usage of static schedulers are fairly limited. To overcome the problem of suboptimal load balancing, tasks are often assigned to processors during run-time. Doing so, however, requires dynamic scheduling, which is in itself a program that has to run alongside the computation. This can become a problem when tasks are small enough and defining an optimal distribution of tasks among workers are nontrivial.

By increasing the number of workers, we expect the total running time to decrease. However, scheduling more and more workers in less and less time becomes increasingly more difficult. Eventually, the scheduling duties will outgrow the computational tasks in complexity and become a severe bottleneck for the application. Unless, that is, the computational burden of the scheduler is also distributed among the working threads, along with the original tasks. Thus, both the total overhead and scheduling speed arising from the parallelization can grow proportionally with the level of parallelization. This way not only the computation, but the parallelization model itself becomes scalable with the number of worker threads.

Standard interthread communication protocols such as event or message based models, shared structures relying on mutual exclusion, locking mechanisms and other heavy parallel constructs can slow down or even halt the execution of certain threads while paving the way for just one. In other words, introducing a new workers to the system might do more harm than good for overall performance, if the complexity of thread management rises faster than the computational gains from higher task throughput.

To battle the above-mentioned problems, we designed a system in which all threads are self-scheduled and managed. Meaning the execution of a single task on a thread must not rely on any activity that might block other threads from doing the same. Furthermore, idle threads must chose available tasks for themselves from a locking-free database, based on information gathered by nonblocking constant time complexity functions.

By following the aforementioned paradigms, the additional overhead from each newly introduced worker can be contained within the new threads themselves, with no possibility of bleeding into existing threads. With the lack of central management and workers having only a loose connection through a catalogue of tasks from which they are free to choose from, this design inherently supports a heterogeneous mixture of workers such as pinned threads for different types of CPUs, GPUs and FPGAs.

### 3.3 Contract Book

Idle workers are obliged to sign up for new tasks. In order to make this endeavor go smoothly, threads can browse a catalogue of available tasks called the Contract Book. This is an object shared among all threads and mostly constitutes of read-only metadata describing the contained tasks. This way workers are not only given new tasks, but tasks that their own scheduling logic finds desirable. Once the preferred task is found, the option can be locked in and made visible for other workers by flipping an atomic boolean based flag, which indicates a task’s availability.

Tasks are organized into larger groups based on their dependency on particular data. Accessing tasks through groups makes task selection faster and can help certain workers—especially those assigned to devices with limited on-board memory—to chew through tasks in an order that guarantees optimal memory management with minimal IO operations between host and device. Other workers might be less lenient to stick to a specific group of tasks. They might go for the largest or smallest of tasks in each group, clear out leftovers or look for other special cases.

In order for workers to be able to choose the most suitable group of tasks, the groups themselves carry metadata akin to contained tasks. In fact, we can view groups as bigger tasks composed by smaller tasks. This also means entire groups can be chosen by a worker via flipping the group’s availability flag. Doing so locks out other workers from choosing new smaller tasks within the group, however, ongoing processes are unaffected and their corresponding smaller tasks will be unavailable for the worker that flipped the group flag.

Given the Contract Book’s locking-free nature, workers are never blocked during task selection. Signing up for a certain job might fail however. Failing to flip an availability flag can only mean another worker has taken the task in the meantime. Thankfully this does not result in a slowdown, because the worker simply skips all operations related to the chosen task and immediately starts looking for the next one. Hence, while introducing new workers into the system might increase tasks being snatched from already present workers, this only results in a speed up and earlier termination of the threads that failed to lock in their selection.

### 3.4 Hash Based Semi-Dynamic Scheduling

So far we were focusing on uniting workers into a loosely connected system with low overhead and locking-free task management devoid of any kind of performance hindering interference between threads. What is left to discuss is how workers can effectively utilize their given autonomy for maximal performance gains. In this section we will primarily focus on GPU based systems, but the presented framework offers enough flexibility to effectively utilize other hardware configurations as well.

On the CPU side of things, the workflow can be made very simple, because in terms of raw theoretical performance even the most powerful CPUs tend to be dwarfed by modern NVIDIA accelerators. There is, however, an addressable problem for CPU based task execution: littering the GPU pipeline with minuscule tasks would lead to significant performance drops. For this reason the CPU based workers are tasked to execute all calculations below a certain threshold of computational complexity. This cutoff point is essentially where the GPU becomes efficient at calculations, while also being small enough for the CPU to finish before the GPU does—and by doing so avoids CPU bottlenecking. Therefore, the optimal value is system dependent, so while that threshold is unknown at compile time, it is always known at runtime, meaning the target complexity for CPU cutoff is known by all workers at all times and can be part of each thread’s built-in static scheduler.

In the current context, by static scheduling we mean scheduling related information that is imprinted into the self-scheduler of every worker object by the object’s own constructor. This information must not change during the object’s life cycle, thus static elements of the schedulers are essentially free of overheads and part of how workers naturally behave without requiring any feedback from the system in which they operate in.

Since a constantly changing scheduling policy would require the worker to track changes or capture meaningful events from the environment, it becomes clear that covering as much scheduling duty as possible by the worker’s much more basic static scheduler is desirable. Extending static coverage beyond basic functionality is possible by creating heuristics that try to guess the task with the highest likelihood of being chosen by a dynamic scheduler—that is aware of other workers and their current situation.

The key to writing such heuristics is predictability. All types of workers are known at compile time and static schedulers are also aware of the CPU/GPU configuration of the current system. By understanding the strategies of all possible worker types and knowing the optimal number of each type for the current hardware, an optimal strategy can be precalculated.

Sticking to this global strategy is effortless as individual workers are oblivious to such strategies and simply pick tasks one by one based on a constant time complexity hash function that seemingly just happens to comply with this global strategy when viewing multiple workers as a team. In other words, workers schedule themselves on an individual level, however, the difference in the order in which workers iterate through tasks leads to spontaneously synergetic behavior between team members.

**Figure 2:**
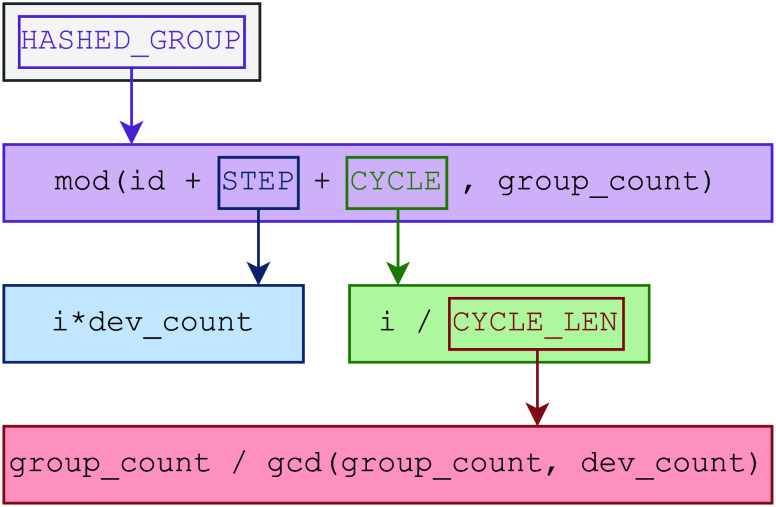
Hash function used for choosing the next group of tasks. Ideal for GPU based workers. Workers initially start with the groups based on their unique identification (id). After the current group has been exhausted, the selection is shifted by the number of GPU based workers (STEP). Once this initial subset of available groups is done, the worker reiterates over the possible groups and will pick one that belongs to an another worker’s initial subset. This is handled by the modulo function (where group_count is the total number of groups). However, reiterating over and over this way does not necessarily make all groups reachable by the worker. We will only generate all possible group indexes if the number of groups and the number of GPU based workers are relative primes. To combat this issue we precalculate the length of the cyclical subset of groups (CYCLE_LEN). Once this value is known, the variable CYCLE will store how many loops we have done so far and we will shift our selection—in addition to shifting from STEP—by the same amount as the value itself. By doing so we ensure different groups are selected in every cycle.

To summarize our findings, a good task selecting hash function (Figure 2) has to follow the following rules:

- Constant time complexity: hashing should be near instantaneous and must not slow down with larger system and model sizes.
- Individual level: always selects one of the most desirable tasks for the worker. Solving a sequence of desirable tasks must yield high performance. For GPU based workers, this mainly boils down to batched matrix operations where the computational complexity is high enough to saturate the device, while at the same time also featuring reoccurring data in subsequent instructions in a way that data already present in device memory can be used multiple times. By doing so, the compute to IO kernel ratio can be increased.
- Team level: taking globally visible tasks is the only possible interaction between workers. A good team player will get rid off tasks undesirable by others and will not ruin a worker’s streak of selecting highly desirable tasks by taking one of the tasks for itself.

CPU based workers find all small tasks equally desirable, hence any order will suffice. This construction is essentially just a parallel *for loop* with dynamic scheduling, meaning tasks are not predistributed, but instead, workers come back again and again to take the next task not yet taken by others.

**Figure 3:**
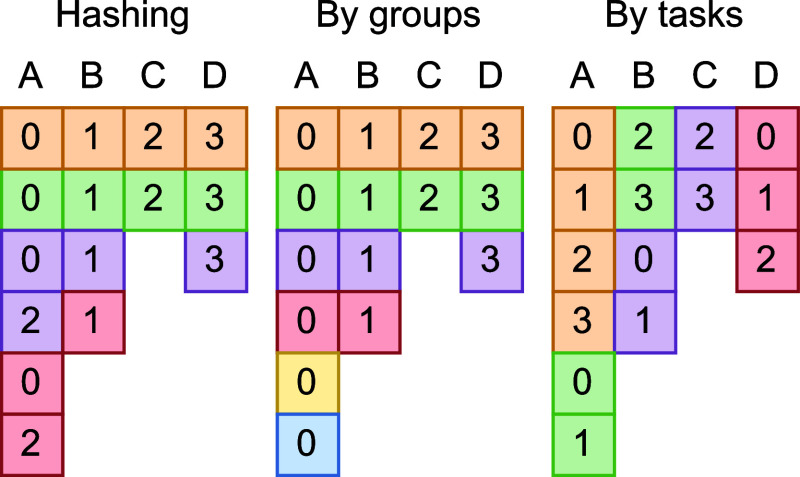
Solving four groups of tasks (A, B, C, and D) with different schedulings. Each task is represented with a square, while the number inside is the unique identifier of the worker that solved the task. For simplicity’s sake, each task takes the exact same time to execute. This way the colors can show which tasks were solved in parallel. Assigning different groups to different workers (scheduling by groups) can result in unwanted idle time due to inconsistencies between the sizes of groups. Ignoring groups and treating the Contract Book as a long queue of tasks (scheduling by tasks) results in high IO overhead due to many devices taking tasks from the same groups. Hashing on the other hand assigns different groups to different workers whenever possible, but at the same time allows multiple devices to work within the same group if necessary.

With smaller tasks out of the picture, all remaining tasks are suitable for GPU accelerators. Their order of execution, however, can make or brake the effectiveness of our strategy. Assigning tasks from different groups in the Contract Book to each worker ensures assigned tasks are undesirable for others, due to different groups requiring different sets of matrices to be loaded in memory (Figure 3). Each GPU moves to a new group after the previous one is exhausted.

Eventually, the number of untouched groups will fall below that of the GPU based workers. This is where hashing truly becomes useful oversimpler constructs such as a parallel for cycle. With hashing we can deliberately cause collisions, meaning multiple workers—despite having different unique IDs—will be assigned to the same group of tasks. By doing so the remaining groups will be finished at an enhanced speed. This way our model can dynamically scale between highest task throughput (focusing on maximum number of solved tasks per given time) and lowest group execution time (focusing on minimizing the time it takes to finish a given group of tasks).

In the initial phase of the computation we can reap the benefits of having statically scheduled disjoint sets of independent jobs, while still having the late-stage option to iron out scheduling imperfections and make runtime optimizations as our complex multiGPU computation progresses. In Figure 2 we show an adequate hashing function, which we also chose for our implementation.

### 3.5 Dedicated IO Streams and Memory Mapping

Instead of relying on NVIDIA’s own virtual memory tables through regular use of *cudaMalloc* and *cudaFree*, we allocate memory only once and use our own model to map memory and enable H2D data transfers. Our solution has been already discussed in ref (21).

Our newest implementation, utilized in the current work, augments performance by executing H2D kernels on desynchronized IO streams during computations. Encapsulation has also been improved as memory tables are now private and all memory management related optimizations are now handled internally, without the compute algorithm having any visibility on how the requested data was produced on the particular device.

### 3.6 Dynamic Memory Buffer Utilization

Locality of reference can be exploited at multiple levels of our computation. First of all, basic arithmetic operations belonging to the same task have exceptionally high spatial data locality due to position dependent index ranges and TTcache’s ability to place data in the order of execution without any gaps in-between elements.^21^ Second, tasks relying on the same set of matrices are grouped together. Finally, during Davidson algorithm, the entire set of groups of tasks might be reused at a later time.

IO operations can be reduced dramatically by increasing the length of GPU buffers to accommodate whole sets of data meant for higher levels of computation. Fixing our model at a level we find reasonable would result in polynomial space complexity. However, there is no benefit to having unused memory, just as we have no use for computation that would exceed our current hardware’s memory limits. Thus, we expect the optimal approach to use all available memory as buffer and dynamically scale between the levels. This way—as matrix sizes grow—the algorithm slowly transforms from an IO call optimized, but memory hungry approach to a space optimized, albeit slower variation.

Helped by the fact that streamed data can be placed into the buffer sequentially, it is effectively effortless to store immutable data from left to right and mutable from right to left. The space in-between can act as a temporary smaller buffer for single-use matrices produced locally on the device. Whenever the two sides get too close, the read-only matrices on the left side can be deregistered and eventually overwritten by newer data. This violates the rule of always keeping ancestral data for TTcache nodes, however since the data set is never modified by the device, it can be recovered from host memory at any point during the worker’s life cycle.

Reconstructing previously deleted data from host memory comes at an IO penalty, however, it only happens when available memory is insufficient. Thus, the IO overhead from redownloads will be based on the level at which deletions are frequent. Multiple deletion without reaching a leaf node indicates that the complete path from the root does not fit into device memory and—while our computation might suffer from high IO overhead due to low level deletes—at the very least we made a computation possible that otherwise would have caused memory overflow.

Also, by omitting deletes based on the traversal of the data dependency tree and solely relying on the preemptive deregistration of immutable data as described above, it is possible to store multiple root to leaf paths in memory at the same time. This further reduces IO calls as different paths are not required to be disjoint sets of data. When memory is sufficient, even the entire data dependency tree can be held in device memory at all times, thus reducing IO calls for subsequent runs to zero.

## 4 Numerical Results

In this section, we present benchmark results obtained via large-scale *SU*(2) spin adapted density matrix renormalization group simulations on selected strongly correlated molecular systems. The F_2_ molecule in CAS(18,18) orbital space,^76^ i.e., 18 electrons on 18 spinful orbitals, was chosen as it corresponds to current limit of exact diagonalization, the nitrogen dimer is often used as a benchmark system as it has a notorious character in chemical bond formation,^77^ and the FeMoco cluster which is in the focus of theoretical chemistry due to its important role in nitrogen fixation–i.e., reduction of nitrogen (N_2_) to ammonia (NH_3_)^78^ – which is essential for the biosynthesis of nucleotides like DNA underlying all life forms on earth. In addition, it also serves as a large benchmark system studied in various previous works.^15,21,79−81^ Since scaling analysis as a function of the number of GPU devices has already been reported in ref (21). here we focus only on the accessible computational power of a single node supplied with eight NVIDIA GPU devices. In addition, as the DMRG matrix and tensor algebra is reformulated according to non-Abelian quantum numbers, the bond dimension *D* stands for the number of renormalized multiplets. When the corresponding *U*(1) bond dimension is also indicated it is denoted explicitly as *D*_*U*(1)_^eff^.

To present precise performance measurements, we determine all matrix/tensor sizes that are involved in a given tensor operation, and the sum of the related elementary additions and multiplications is divided by total elapsed time during execution (also including allocation, IO overheads etc.). Here we also guide interested readers to ref (65). where even detailed scaling analysis of computational complexity related to DMRG matrix product operator (MPO) bond dimension and matrix product state (MPS) bond dimension is presented and theoretical scaling laws are validated by numerical simulations on two-dimensional quantum lattice models.

**Figure 4:**
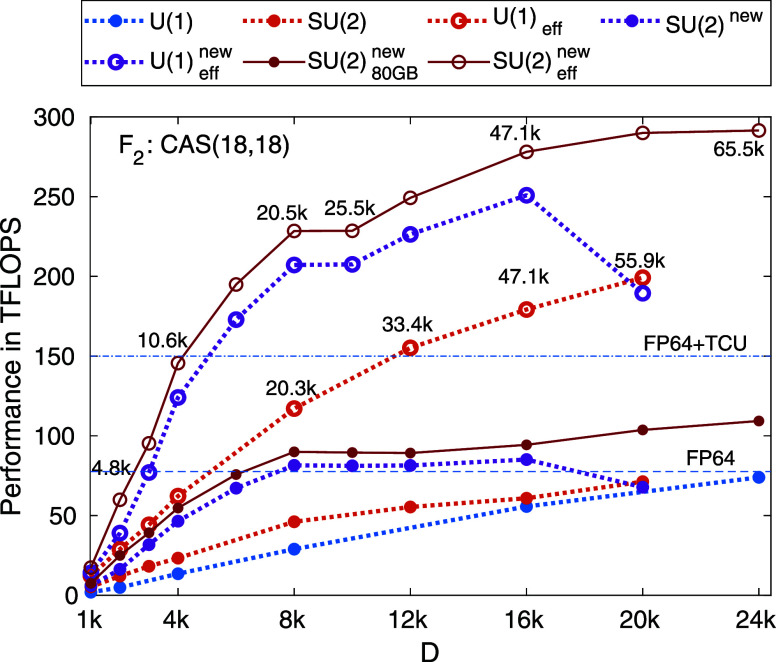
Benchmark results obtained via the SU(2) spin adapted hybrid CPU-multiGPU DMRG for the F_2_ molecule for a CAS(18,18) orbital space. Calculations have been performed on a dual AMD EPYC 7702 CPUs with 2 × 64 cores compiled with eight NVIDIA A100-SXM4–40GB devices. The estimated FP64 theoretical upper bound for eight NVIDIA A100-PCIE-40GB GPU devices is shown by the dashed line while the same but also including highly specialized tensor core units (TCUs) by the dashed-dotted line. The blue and red curves show the performance measured in TFLOPS for parallelization model presented in ref (21) using *U*(1) and *SU*(2) symmetries, respectively. Results obtained via our new parallelization model, Hash Driven Semi-Dynamic scheduling, NVIDIA, is shown by dark purple color. The lower bound on the effective performance in terms of *U*(1) bond dimension after rescaling are shown by open symbols. Numbers next to the data points show the measured largest *D*_*U*(1)_ bond dimension values corresponding to *SU*(2) bond dimension, *D*. Data points connected by solid line correspond to calculations performed on a dual AMD EPYC 7742 CPUs with 2 × 64 cores compiled with eight NVIDIA A100-SXM4–80GB devices.

**Figure 5:**
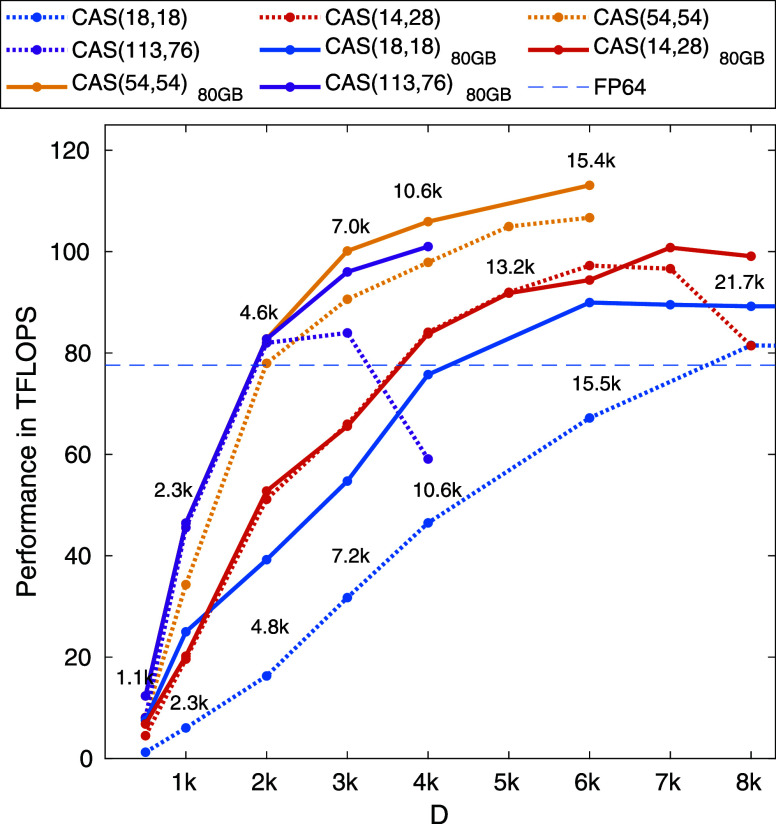
Benchmark results obtained via the SU(2) spin adapted hybrid CPU-multiGPU DMRG for the F_2_ N_2_ and FeMoco molecular systems for CAS(18,18), CAS(14,28), CAS(54,54) and CAS(113,76) orbital spaces. Calculations have been performed on a dual AMD EPYC 7702 CPUs with 2 × 64 cores compiled with eight NVIDIA A100-SXM4–40GB devices. The estimated FP64 theoretical upper bound for eight NVIDIA A100-PCIE-40GB GPU devices is shown by the dashed line. Data points connected by solid line correspond to calculations performed on a dual AMD EPYC 7742 CPUs with 2 × 64 cores compiled with eight NVIDIA A100-SXM4–80GB devices.

Regarding hardware specifications, IO communication and size of VRAM are important issues. All calculations presented in this work are performed on a single A100 based node. For most of the calculations in an A100 node the accessible size of VRAM is limited to 8 × 40GB. To highlight importance of the size of VRAM, the performance measurements have also been carried out on a node with 8 × 80GB VRAM (see Figures 4 and 5). Data point presented for our largest bond dimension in Table 3 was obtained on this latter hardware configuration.

### 4.1 Boosting the Effective Performance via Non-Abelian Symmetries

From technical point of view, utilization of non-Abelian symmetries leads to lower memory demands for a given accuracy threshold, denser sector decomposition of the operators and increased number of tasks, which are advantageous for massive parallelization. However, the generation of the independent tasks to be performed for the underlying algebra requires more delicate mathematical framework as discussed in Section 2. In Figure 4 we summarize our benchmark results obtained via the *SU*(2) spin adapted hybrid CPU-multiGPU DMRG for the F_2_ molecule for a CAS(18,18) orbital space. This relatively small CAS space corresponds to current limit of exact diagonalization, thus our results demonstrate that the exact wave function can be determined for high accuracy in fraction of time via DMRG on GPUs. Calculations have been performed on dual AMD EPYC 7702 CPUs with 2 × 64 cores complemented with eight NVIDIA A100-SXM4–40GB devices. To highlight performance dependence on GPU memory size some selected calculations have also been repeated on dual AMD EPYC 7742 CPUs with 2 × 64 cores complemented with eight NVIDIA A100-SXM4–80GB devices (see data points connected by solid lines). The estimated FP64 theoretical upper bound for eight NVIDIA A100-PCIE-40GB GPU devices^82^ is shown by the horizontal dashed line while the same but also including highly specialized AI accelerators via the tensor core units (TCUs) by the horizontal dashed-dotted line. The blue and red curves show the performance measured in TFLOPS using *U*(1) and *SU*(2) symmetries via implementation presented in ref (21), respectively. These correspond to the largest performance values measured via the Davidson procedure, i.e., the sum of all the computational complexity (CC) given in TFLOP to perform the sequence of matrix and tensor operations according to eq 2 divided by the elapsed time measured in seconds. Therefore, this is an average across the most performant segment of the computation, thus peak performance can be expected to be even higher. It is remarkable that higher performance is measured for a given *D* value in case of *SU*(2) and it reaches the FP64 limit slightly faster as a function of *D* than the *U*(1) counterpart. Therefore, no additional overhead appears for matrix and tensor operations in case of the non-Abelian version.

As the spin of the renormalized basis states is known, the corresponding effective *U*(1) bond dimension, *D*_*U*(1)_^eff^ can be calculated and monitored. In addition, having the performance measurements via the *U*(1) implementation in hand, the ratio between the performance for a given *D* and the corresponding *D*_*U*(1)_ can be estimated. In Table 1 the matching data sets are collected to provide more insights on these quantities. The ratio *R*_CC_ could be used to rescale the measured *SU*(2) performance to predict the effective performance in case of a *U*(1) implementation only to reach the same accuracy. This, however, cannot be determined for large *D* values. Since the ratio between *D* and *D*_*U*(1)_^eff^, *R*_D_, is always smaller by at least a factor of 2 than *R*_CC_ we could use it to provide a lower bound on the effective performance. After rescaling the measured *SU*(2) performance with *R*_*D*_ one arrives to the curve shown by open symbols. Numbers next to the data points indicate the corresponding largest *D*_*U*(1)_^eff^ bond dimension values. Thus, this curve is an estimate of the lower bound of the effective performance in terms of *U*(1) bond dimension, i.e., what *U*(1) bond dimension should be used to achieve the same accuracy and what performance would such calculation correspond to. In general, we conclude that our framework presented for *U*(1) symmetry in ref (21). can be utilized equally for non-Abelian symmetries, like *SU*(2) spin symmetry, without introducing additional overhead.

**Table 1:** Collected Data Sets to Provide an Estimate on the Ratio, *R*_CC_, between the Largest Performance Measured for a Given *D* and *D*_*U*(1)_, and the Ratio, *R*_D_, between *D* and *D*_*U*(1)_^eff^^a^

*D*	C*C*_max_ in GFLOP	*D*_*U*(1)_^eff^	*D*_*U*(1)_	C*C*_max_ in GFLOP	*R*_CC_	*R*_D_
1024	680	2361	2048	1459	2.15	2.30
2048	4196	4910	5120	20,656	4.92	2.40
4096	27,586	10,928	10,240	160,912	5.83	2.67
8192	193,949	20,797	20,480	1,166,565	6.01	2.54

a Here, C*C*_max_ stands for the largest total computational complexity measured in FLOP for a given Davidson step.

**Table 2:** Fitted Exponents for the Eight GPU Accelerated Diagonalization Step for the F_2_, N_2_ and the FeMoco Molecular Systems for Various DMRG Parameters for Data Sets Shown in Figure 6^a^

system	CAS	γ_1_	γ_2_
F_2_	(18,18)	1.11	3.10
N_2_	(14,28)	1.15	3.34
FeMoco	(54,54)	1.10	3.33
FeMoco	(113,76)	1.01	-

a Exponent γ_1_ corresponds to data sets measured for performance up to the FP64 limit and γ_2_ to data sets with saturation in performance above the FP64 limit.

### 4.2 Boosting Performance via New Algorithmic Model for Parallelization

In ref (21). we have already shown the monotonic increase in the performance as a function of the bond dimension for all selected systems, but the actual speedup utilizing GPU acceleration and its dependence on the number of GPU devices are highly system dependent (see Figure 7 in ref (21).). Since the same accuracy can be reached with significantly lower *D* values in case of *SU*(2) symmetry it is advantageous to improve the performance for the smaller *D* regime as well. Although, the implementation reported in ref (21). works equally well for *U*(1) and *SU*(2) symmetries as discussed in the previous section (see Figure 4) the major speedup was measured for relatively larger bond dimensions. Therefore, we have designed and developed a new mathematical model presented in Section 3 as a flexible framework for various types and stages of parallelization, including MPI (multiple nodes), CUDA (multiple GPUs) and classical threading (multiple CPUs). The concurrent utilization of all physical CPU cores and NVIDIA accelerators residing within a local node is made possible by our in-house designed C++ based threading, lock-free interthread communication protocols and custom virtual device-memory mapping models.

Here we demonstrate the drastic increase in performance utilizing our novel solution for the F_2_ molecule in the CAS(18,18) orbital space. The measured performance as a function of *D* is shown in Figure 4 by the dark purple curve. The performance in most of the cases doubled at least, leading to a factor of 2 to three further reduction in computational time. For *D* ≥ 8192, even together with the IO overhead, the measured performance is above the FP64 theoretical limit, indicating that NVIDIA Tensor Cores can also be utilized by our new parallelization model (alongside the conventional FP64 units). Most remarkably, however, another main result is the dramatic increase in performance for smaller bond dimension values as is apparent in Figure 4. In fact, the FP64 limit is almost reached by *D* = 6144 corresponding to *D*_*U*_(1) = 15,755. We also confirmed that the measured largest computational complexity via the new version is withing two percent agreement with data collected in Table 1. After resealing via *R*_*D*_, the lower bound of the effective performance in terms of *U*(1) symmetries, shown by the open dark purple symbols, is already above 250 TFLOPS while the actual value using *R*_CC_ could be close to 500 TFLOPS. This demonstrates again the tremendous increase in performance utilizing non-Abelian symmetries via our new model for parallelization.

For *D* = 20,480, however, a breakdown in performance is observed (see Figure 4). This is due to the fact that for such large *D* value the 40GB of VRAM for a single GPU device is not enough to store a meaningful amount of data, resulting in poor cache performance, which ultimately will lead to much higher IO overhead. The inability to keep data in cache even at lower levels of data abstraction, as explained in Section 3, will likely cripple performance. Recall that in our new model we use the GPU memory more aggressively to boost the performance while for very large *D* values, when GPU memory is not sufficient, a higher price in performance is paid. To simulate such scenario we have performed tests by limiting the available GPU memory to 5, 10, 15, 20, 25 GB and confirmed the systematic decrease in the number of H2D IO calls and the dramatic increase in performance. In fact, repeating the calculations for bond dimension up to *D* = 24 k, but using A100 GPU devices with 80GB of VRAM, the observed breakdown in performance can be lifted and even fully eliminated (see data points connected with solid red line.) Fortunately, the unfavorable loss in performance can also be improved or even omitted utilizing the NVIDIA fast D2D NVLINK connection allowing the algorithm to treat the memory of the eight GPU devices as a single big memory unit (in the current case corresponding to 320 GB). Implementation details of such highly sophisticated kernel keeping track of all data segments already available in VRAM of the individual devices or still required to be transferred from the host is under development and will be part of a subsequent work.

It is important to mention, that for *D* = 24,576, i.e., for *D*_*U*(1)_ = 65,536 the exact solution, the so-called full-CI limit, is recovered. This is basically the current limit of exact diagonalization on HPC infrastructures. Such very large *D*_*U*(1)_ = 2^16^ value is also the largest one utilized via Google’s tensor product units (TPUs), but in ref (19). the simulations have been performed for a noninteracting spinless Fermion model system. In contrast to that, here we present a fully interacting ab initio simulation via *SU*(2) spin symmetry reaching an effective *U*(1) performance close to 300 TFLOPS as is shown by data points with open symbols connected by a solid line.

In order to demonstrate that our non-Abelian symmetry related algorithmic solutions and the new technical developments are not limited to small CAS spaces in Figure 5 we present benchmark results for much larger CAS spaces for the nitrogen dimer and for the FeMoco molecular cluster. It is apparent that increasing the number of orbitals a much faster increase in performance can be obtained as a function of bond dimension. This is due mainly to the new SBGEMMs protocol discussed in Section 3 since for each sector decomposed algebra the computational complexity increases with *N*^2^. For the FeMoco molecular cluster for the CAS(54,54) orbital space^79^ the measured maximum performance is around 108 TFLOPS, being again well above the FP64 limit. For CAS(113,76) orbital space^80^ similar results are obtained, however, the limitation of the 40GB VRAM size appears for smaller *D* values. Therefore, the breakdown in performance is experienced already for *D* = 4096 (see Figure 5). This can, however, be lifted again over 100 TFLOPS when GPU devices with 80GB of VRAM is utilized as is shown by data points connected by a solid line. In addition, the breakdown in performance can be shifted to much larger *D* values via the utilization of the fast D2D NVLINK communication. Here we remark that the performance and required VRAM heavily depend on the number of the quantum number sector based tasks and on the system size, *N*. The effect of such highly nontrivial function is clearly reflected by the different curves shown in Figure 5.

### 4.3 Diagonalization Time Including IO Overhead versus Bond Dimension

**Figure 6:**
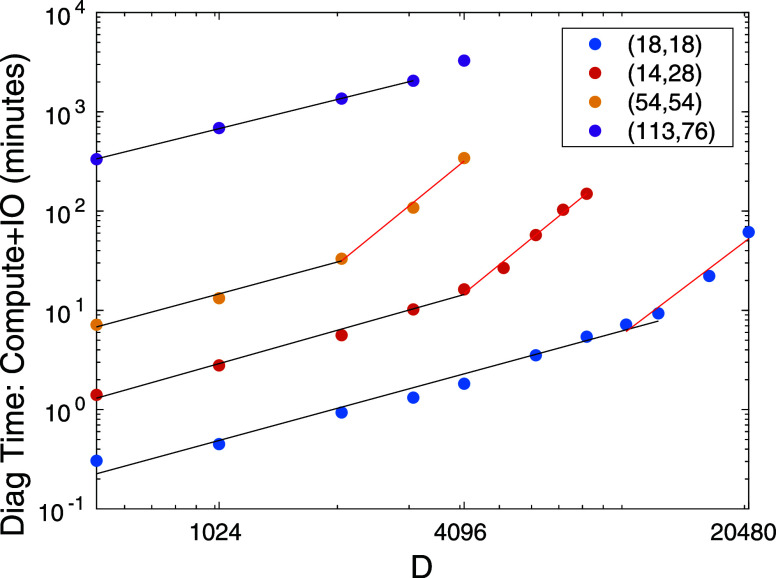
Total time of seven DMRG sweeps for the eight GPU accelerated diagonalization procedure measured in minutes including IO overhead for the F_2_ CAS(18,18), N_2_ CAS(14,28), FeMoco CAS(54,54) and CAS(113,76) as a function of DMRG bond dimension. The solid lines are results of first-order polynomial fits on selected data sets corresponding to measured performance up to the FP64 limit (black) and for performance above the FP64 limit (red). The fitted exponents are summarized in Table 2.

Completing the performance analysis as a function of the bond dimension, here we repeat the same, but for the computational time spent on the diagonalization of the effective Hamiltonian (see also ref (21).). The scaling of the total time spent on the eight GPU accelerated diagonalization of the effective Hamiltonian including H2D and D2H IO measured for seven DMRG sweeps is presented in Figure 6 on a double logarithmic scale as a function of DMRG bond dimension for the F_2_, N_2_ and FeMoco cluster for CAS(18,18), CAS(14,28), CAS(54,54) CAS(113,76) orbital spaces. The solid lines are results of first-order polynomial fits on selected data sets corresponding to calculations with performance up to the FP64 limit (black) and for *D* values when saturation in performance is reached together with memory clearing on the GPU devices and redundant H2D IO steps (red). In the following, the two kind of exponents are denoted by γ_1_ and γ_2_.

For F_2_ the exponent is estimated to be γ_1_ = 1.11 when no clearing step is required, while it increases to γ_2_ = 3.1 when saturation is reached and clearing together with redundant H2D IO protocol is utilized. For the larger orbital space, i.e. for N_2_, the linear scaling is again recovered with exponent γ_1_ = 1.15 for *D* ≤ 4096 while γ_2_ = 3.3 is obtained for larger *D* values due to the saturation in performance. We remark that with *D* = 8192 the ground state reference energy obtained by a sixth order Coupled Cluster calculation (CCSDTQPH), reported in ref (77). is also reproduced with 6 digit accuracy.

For the FeMoco cluster for CAS(54,54) γ_1_ = 1.1 for *D* ≤ 2048 while again a much larger value, γ_2_ = 3.3, is obtained when the saturation in performance is reached for *D* ≥ 2048. Here we note that in order not to waste computational resources, the calculations with *D* = 5120 and 6144 were stopped after the fourth sweep as performance measurements were already completed at that iteration stage and the saturation of the curve in Figure 5 together with the cubic scaling already obtained for the range 2048 ≤ *D* ≤ 4096 would not be effected. For the FeMoco cluster for CAS(113,76) γ_1_ = 1.01 up to *D* = 3072 while again a much larger value for γ_2_ is expected for *D* ≥ 4096 values.

To provide further insights, in Table 3 the measured total diagonalization time including IO overhead are collected for various parameter sets. This data set heavily depends on the total number of Davidson steps, thus both algorithmic improvements and hardware configuration can lead to further reduction in diagonalization time. Here we also remark that the *SU*(2) spin adapted variant of the dynamically extended active space (DEAS) procedure^75^ is not developed yet. Therefore, for providing fair comparison with our previously published *U*(1) results the total execution time is measured between the second and ninth sweep, in order to exclude the time of the nonoptimized warmup procedure. This in general would have provided some 5–10% overhead. In addition, we observed that due to the lack of the optimized warmup a factor of 2 to three higher number of matrix vector multiplications were utilized by the Davidson algorithm for the same convergence during the third and fourth sweeps. These drawbacks will be eliminated via the *SU*(2) adapted DEAS and RAS procedures^75,83^ that are also part of our current developments.

**Table 3:** Total Computation Time Together with IO Overhead for the Eight GPU Accelerated Diagonalization Step for the F_2_, N_2_ and the FeMoco Molecular Systems for the Spin Adapted DMRG for Seven Sweeps and for Various Bond Dimension Values^a^

CAS	*D*	effective *D*_*U*(1)_	#of sweeps	diag time: compute + IO
(18,18)	4096	10,932	7	1.8 min
(18,18)	20,480	57,221	7	61 min*
(18,18)	24,576	65,536	7	63.5 min^†^
(14,28)	4096	11,226	7	16.3 min
(14,28)	8192	22,243	7	2.5 h*
(54,54)	1024	2256	7	13.25 min
(54,54)	2048	4642	7	33.11 min
(54,54)	3072	7061	7	1.8 h
(54,54)	4096	10,917	7	5.7 h
(113,76)	512	1502	7	5.6 h
(113,76)	1024	3426	7	11.3 h
(113,76)	2048	6965	7	22.7 h
(113,76)	3072	9058	7	34.2 h
(113,76)	4096	12,101	7	54.6 h*

a The largest measured effective *U*(1) bond dimension is also displayed. * indicates calculations with increased execution time due to breakdown in performance via our current implementation facing memory limitation of the individual GPU devices. ^†^ refers to calculations on the A100 node with 8 × 80GB VRAM.

### 4.4 Renormalization Procedure

As in case of our previous parallelization scheme presented for the U(1) symmetry in ref (21), the performance of the multiGPU variant of the renormalization procedure does not scale optimally with the number of devices for non-Abelian symmetry as well. This is due again to the heavy D2H and H2D IO demands as the memory requirement to store the renormalized operators scales with *N*^2^. In fact, for small and intermediate bond dimensions the IO overhead can be larger than the computational gain when several GPU devices are utilized, except for very large bond dimension values. Compared to the CPU-only limit, however, even a singe GPU accelerated renormalization procedure becomes a factor of two to four faster. Therefore, in most of the calculations we have used a single GPU device during the renormalization step. Again a much more efficient renormalization algorithm will become available by utilizing the fast NVIDIA D2D communication immediately reducing the redundant H2D IO significantly. Fortunately, due to the general structure of the parallelization model discussed in Section 3 a single framework is developed for the various algorithmic tasks, therefore, improvements obtained for the diagonalization procedure via fast D2D communication will become immediately available for the renormalization procedure as well. Further details will be presented in a subsequent work.

## 5 Conclusions

In this work, we have presented novel algorithmic solutions together with implementation details to exploit the computational power of AI accelerators and extend current limits of tensor network state algorithms via non-Abelian symmetries on high performance computing infrastructure. Building on state-of-the-art hardware and software technologies these includes the following main contributions:

- We have introduced a new decentralized threading model, where newly launched threads put no strain on their pre-existing brethren, as all overhead of parallelism is self-contained. In our newly designed system all threads are self-scheduled and make decisions autonomously. Threads are guaranteed to be able to work independently and are only loosely connected via a globally visible lock-free construct we call the Contract Book. Therefore, the computational burden of parallelization remains marginal and evenly distributed among workers, even at high thread counts.
- A new hash based semidynamic scheduling protocol has been developed, enabling our model to dynamically scale between highest task throughput and lowest task group execution time. In the initial phase of the computation we can reap the benefits of having statically scheduled disjoint sets of independent jobs, while still having the late-stage option to iron out scheduling imperfections and make runtime optimizations.
- We have decomposed the sequence of position dependent matrix arrays (renormalized operators) into subgroups. Our custom tailored virtual memory management ensures this data is mapped into device memory space in accordance with the order of execution. The arising high spatial data locality is exploited through the use of specific sequences of strided batched matrix operations. A significantly higher level of SIMD parallelization has been reached for the overall multiplication of the entire matrix array, since the matrices residing in these subgroups can be numerous.
- In order to reduce IO operations dramatically, an adaptive buffering technique is used to dynamically match the level of data abstraction to system resources. A computation that normally would have failed due to insufficient memory will now become runnable by the frequent deregistration of data at lower levels of abstraction. This reduces the effective level at which cache operates. The inverse happens when available memory is abundant. Deregistrations will occur at a higher level, leading to more efficient caching. Ultimately, memory demand at a given time will be mapped to available memory at a given time. For every point in time, an elevation in memory usage will yield higher efficiency in caching, while drops in memory demand will cost us in performance. In our adaptive model the actual memory demand will match global device memory (VRAM) at all times. Instead, it is the cache performance that is variable.
- We have presented a general algorithmic design to separate the Clebsch-Gordan layer from the MPS layer for non-Abelian symmetries which allows efficient execution of the underlying algebra without additional overhead. As a result, the tensor and matrix algebra developed for *U*(1) symmetry is modified only by constant multiplication factors that can be precalculated and stored in lookup tables.
- We have performed benchmark calculations up to *U*(1) bond dimension *D* = 2^16^ and on Hilbert space dimensions up to 2.88 × 10^36^ obtained via the large-scale *SU*(2) spin adapted hybrid CPU-multiGPU density matrix renormalization group method for the F_2_, N_2_ and FeMoco strongly correlated molecular systems. These demonstrate the utilization of the highly specialized AI accelerators via NVIDIA tensor core units (TCUs), leading to a performance around 115 TFLOPS on a single node supplied with eight NVIDIA A100 devices. The effective performance to reach the same accuracy with a *U*(1) implementation only was estimated to be in the range of 350–500 TFLOPS.
- We have analyzed the total computation time including IO overhead for the diagonalization procedure as a function of DMRG bond dimension and found two sets of exponents determining the overall scaling. For bond dimension values where the obtained performance is not exceeding the FP64 saturation limit the exponent is one leading to linear scaling. On the other hand, when a saturation or even a breakdown in performance is measured a cubic dependence is found as a function of bond dimension. Note that the former behavior has already been reported in ref (21). for an implementation including *U*(1) symmetry only, while the latter regime could have been accessed only via non-Abelian symmetry, due to the big reduction in memory and the tremendous increase in computational complexity.

Our new parallelization model has not only lead to significant increase in maximum performance, but also boosts the performance for small and intermediate bond dimensions. This, together with the tremendous developments in IT-technology, like NVIDIA’s latest Grace Hopper and Blackwell hardware platforms, can raise the non-Abelian variant of the hybrid CPU-multiGPU DMRG to become a routinely applied daily method in simulation of quantum systems for a broad range of disciplines. In fact, a recent application on two-dimensional quantum lattice models reports several orders of magnitude reduction in computational time.^65^

Our developments can also be further improved by utilization of NVIDIA fast D2D communication allowing us to treat the total memory of the individual devices as a single unit, and thus to reduce IO overhead significantly. In addition, combination of the hybrid CPU-multiGPU solution with our message passing interface (MPI) based code to achieve multiNode-multiGPU version of the algorithm will raise its performance to the petascale regime. Therefore, breaking the current computational limits on complex problems that are beyond the scope of conventional methods is becoming a reality.

## Supporting Information Available

Overview of the structure of the operator-table for the kinetic term in eq 1 for the two-site DMRG, i.e., how operators are distributed among the DMRG subsystems; possible derivation of the correction factor, *C̃*, that appears in the matrix and tensor algebra for SU(2) non-Abelian symmetry relying on a Matlab implementation of the Wigner-9j and Wigner-6j formalism (PDF)
