# Supplementary material for: Tensor Network State Algorithms on AI Accelerators
Source: J Chem Theory Comput. 2024 Oct 14;20(20):8897–910. doi: 10.1021/acs.jctc.4c00800 (PMC11500410; doi:10.1021/acs.jctc.4c00800)
Supplement: Supplementary file 1 — ct4c00800_si_001.pdf [file ct4c00800_si_001.pdf]

Supporting Information for Publication: Tensor network state algorithms on AI accelerators

Andor Menczer[1,2] and Örs Legeza[1,3,*]

[1] *Strongly Correlated Systems "Lendület" Research Group, Wigner Research Centre for Physics, H-1525, Budapest, Hungary*
[2] *Eötvös Loránd University, Pázmány Péter Sétány 1/C, 1117 Budapest, Hungary*
[3] *Institute for Advanced Study, Technical University of Munich, Lichtenbergstrasse 2a, 85748 Garching, Germany*

(Dated: September 17, 2024)

In this supporting information for publication, on the one hand, we briefly overview the structure of the operator-table for the kinetic term in Eq. (1) for the two-site DMRG, i.e., how operators are distributed among the DMRG subsystems. On the other hand, we present a possible derivation of the correction factor, $\tilde{C}$, that appears in the matrix and tensor algebra for SU(2) non-Abelian symmetry relying on a Matlab implementation of the Wigner-9j and Wigner-6j formalism.

I. STRUCTURE OF THE OPERATOR-TABLE

In this section, as an example, we briefly overview the structure of the operator-table for the kinetic term in Eq. (1) for the two-site DMRG, i.e., how operators are distributed among the four subsystems, labeled by l, s_1, s_2 and r, respectively. This is shown in Table. I where I stands for the identity operator, P for the phase operator and

	l	s_1	s_2	r
1	$O(2)$	I	I	I
$\sum_{j\beta}$	$O(1)_{j\beta}$	$c_{j\beta}$	I	I
$\sum_{j\beta}$	$O(1)_{j\beta}$	P	$c_{j\beta}$	I
$\sum_{j\beta}$	$O(1)_{j\beta}$	P	P	$c_{j\beta}$
1	I	$O(2)$	I	I
$\sum_{j\beta}$	I	$O(1)_{j\beta}$	$c_{j\beta}$	I
$\sum_{j\beta}$	I	$O(1)_{j\beta}$	P	$c_{j\beta}$
1	I	I	$O(2)$	I
$\sum_{j\beta}$	I	I	$O(1)_{j\beta}$	$c_{j\beta}$
1	I	I	I	$O(2)$

TABLE I. Operator-table for the kinetic term in Eq. (1) for the two-site DMRG with $i \leq j$, i.e., how operators are distributed to four subsystems, labeled by l, s_1, s_2 and r, respectively. I stands for the identity operator, P for the phase operator and O is defined in Eq. (S1). The first column indicates if an external summation is required.

$$
\begin{aligned}
O(2) &= \sum_{ij\alpha\beta} T_{ij}^{\alpha\beta} c_{i\alpha}^{\dagger} c_{j\beta}\,, \\
O(1)_{j,\beta} &= \sum_{i\alpha} T_{ij}^{\alpha\beta} c_{i\alpha}^{\dagger}\,.
\end{aligned}
\tag{S1}
$$

The range of the summation indices i, j in Eq. (S1) lies in the given subsystem, while the range of external summation indicated in the first column of Table. I is determined by the auxiliary subsystem. Thus the overall original scaling with $\mathcal{O}(N^2)$ is reduced to $\mathcal{O}(N)$. For further details we guide the readers to Refs. [1–9].

II. DERIVATION OF THE CORRECTION FACTORS

In this section we present a possible derivation of the correction factor, $\tilde{C}$, that appears in the matrix and tensor algebra for SU(2) non-Abelian symmetry relying on a Matlab implementation of the Wigner-9j and Wigner-6j formalism [10–12], i.e.,

$$
\begin{aligned}
\tilde{C} =& \sqrt{(2j_1'+1)(2j_2'+1)(2j+1)(2k+1)} \times \\
& W_{9\mathrm{j}}(j_1, j_2, j, k_1, k_2, k, j_1', j_2', j')
\end{aligned}
\tag{S2}
$$

where

$$
\begin{aligned}
& W_{9\mathrm{j}}(j_1, j_2, j, k_1, k_2, k, j_1', j_2', j') = \\
& \sum_{x=x_{\min}}^{x_{\max}} (-1)^{2x}(2x+1)\cdot W_{6\mathrm{j}}(j_1, j_2, j, k, j', x) \times \\
& W_{6\mathrm{j}}(k_1, k_2, k, j_2, x, j_2') \cdot W_{6\mathrm{j}}(j_1', j_2', j', x, j_1, k_1),
\end{aligned}
\tag{S3}
$$

with

$$
\begin{aligned}
x_{\min} &= \max(j_1 - j', j_2 - k, k_1 - j_2'), \\
x_{\max} &= \min(j_1 + j', j_2 + k, k_1 + j_2'),
\end{aligned}
\tag{S4}
$$

$$
\begin{aligned}
&|j_1 - j_2| \leq j \leq j_1 + j_2, && |k_1 - k_2| \leq k \leq k_1 + k_2, \\
&|j_1' - j_2'| \leq j' \leq j_1' + j_2', && |j_1 - k_1| \leq j_1' \leq j_1 + k_1, \\
&|j_2 - k_2| \leq j_2' \leq j_2 + k_2, && |j - k| \leq j' \leq j + k,
\end{aligned}
\tag{S5}
$$

and

$$
\begin{aligned}
& W_{6j}(a,b,c,d,e,f) = \tilde{g}(a,b,c)\tilde{g}(c,d,e)\tilde{g}(a,e,f)\tilde{g}(b,d,f) \times \\
& \sum_{n=n_{\min}}^{n_{\max}} \big((-1)^n (n+1)!\big) / \big((n-\tilde{n}_2)! \cdot (n-\tilde{n}_3)! \cdot (n-\tilde{n}_4)! \times \\
& (n-\tilde{n}_5)! \cdot (\tilde{n}_6 - n)! \cdot (\tilde{n}_7 - n)! \cdot (\tilde{n}_8 - n)!\big),
\end{aligned}
\tag{S6}
$$

* legeza.ors@wigner.hu

with

$$\begin{aligned}
&\tilde{n}_1 = 0, && \tilde{n}_2 = a+b+c,\\
&\tilde{n}_3 = c+d+e, && \tilde{n}_4 = a+e+f,\\
&\tilde{n}_5 = b+d+f, && \tilde{n}_6 = a+b+d+e,\\
&\tilde{n}_7 = a+c+d+f, && \tilde{n}_8 = b+c+e+f,\\
&n_{\min} = \max_{i=1\ldots5} \tilde{n}_i, && n_{\max} = \min_{i=6\ldots8} \tilde{n}_i \qquad \text{(S7)}\\
&|a-b| \le c \le a+b, && |c-d| \le e \le c+d,\\
&|a-e| \le f \le a+e, && |b-d| \le f \le b+d,\\
&\mathrm{mod}(a+b+c,1) = 0, && \mathrm{mod}(c+d+e,1) = 0,\\
&\mathrm{mod}(a+e+f,1) = 0, && \mathrm{mod}(b+d+f,1) = 0,
\end{aligned}$$

and

$$\begin{aligned}
\tilde{g}(a,b,c) =& \sqrt{(a+b-c)!(a-b+c)!(-a+b+c)!}/\\
&\sqrt{(a+b+c+1)!}. \qquad \text{(S8)}
\end{aligned}$$

Here we remark that derivation of W_{9j} and W_{6j} is also possible via directly from the Clebsch-Gordan coefficients[10, 13, 14].

[1] Xiang, T. Density-matrix renormalization-group method in momentum space. *Phys. Rev. B* **1996**, *53*, R10445–R10448.

[2] White, S. R.; Martin, R. L. Ab initio quantum chemistry using the density matrix renormalization group. *The Journal of Chemical Physics* **1999**, *110*, 4127–4130.

[3] Szalay, Sz.; Pfeffer, M.; Murg, V.; Barcza, G.; Verstraete, F.; Schneider, R.; Legeza, Ö. Tensor product methods and entanglement optimization for ab initio quantum chemistry. *Int. J. Quantum Chem.* **2015**, *115*, 1342–1391.

[4] Schollwöck, U. The density-matrix renormalization group. *Rev. Mod. Phys.* **2005**, *77*, 259–315.

[5] Schollwöck, U. The density-matrix renormalization group in the age of matrix product states. *Annals of Physics* **2011**, *326*, 96 – 192, January 2011 Special Issue.

[6] Noack, R. M. Diagonalization- and Numerical Renormalization-Group-Based Methods for Interacting Quantum Systems. AIP Conference Proceedings. 2005.

[7] Chan, G. K.-L.; Dorando, J. J.; Ghosh, D.; Hachmann, J.; Neuscamman, E.; Wang, H.; Yanai, T. In *Frontiers in Quantum Systems in Chemistry and Physics*; Wilson, S., Grout, P. J., Maruani, J., Delgado-Barrio, G., Piecuch, P., Eds.; Progress in Theoretical Chemistry and Physics; Springer: Netherlands, 2008; Vol. 18.

[8] Orús, R. A practical introduction to tensor networks: Matrix product states and projected entangled pair states. *Annals of Physics* **2014**, *349*, 117 – 158.

[9] Baiardi, A.; Reiher, M. The density matrix renormalization group in chemistry and molecular physics: Recent developments and new challenges. *The Journal of Chemical Physics* **2020**, *152*, 040903.

[10] D. A Varshalovich, A. N. M.; Khersonskii, V. K. *Quantum Theory of Angular Momentum*; World Scientific, 1988.

[11] Kobi, http://www.mathworks.com/matlabcentral/fileexchange/20619 **2011**,

[12] Weisstein, E. Triangular Inequalities. http://mathworld.wolfram.com/TriangularInequalities.html

[13] Messiah, A. *Quantum Mechanics, Volume II*; North Holland Publisher Company, Amsterdam, 1962.

[14] Werner, M. A.; Moca, C. P.; Legeza, Ö.; Zaránd, G. Quantum quench and charge oscillations in the SU (3) Hubbard model: A test of time evolving block decimation with general non-Abelian symmetries. *Physical Review B* **2020**, *102*, 155108.
